# Transcription factors regulating the specification of brainstem respiratory neurons

**DOI:** 10.3389/fnmol.2022.1072475

**Published:** 2022-11-29

**Authors:** Yiling Xia, Ke Cui, Antonia Alonso, Elijah D. Lowenstein, Luis R. Hernandez-Miranda

**Affiliations:** ^1^The Brainstem Group, Institute for Cell Biology and Neurobiology, Charité Universitätsmedizin Berlin, Corporate Member of Freie Universität Berlin and Humboldt-Universität zu Berlin, Berlin, Germany; ^2^Functional Genoarchitecture and Neurobiology Groups, Biomedical Research Institute of Murcia (IMIB-Arrixaca), Murcia, Spain; ^3^Department of Human Anatomy and Psychobiology, Faculty of Medicine, University of Murcia, Murcia, Spain; ^4^Developmental Biology/Signal Transduction, Max Delbrück Center for Molecular Medicine, Berlin, Germany

**Keywords:** transcription factors, brainstem development, progenitor domains, neuronal specification, respiratory neurons

## Abstract

Breathing (or respiration) is an unconscious and complex motor behavior which neuronal drive emerges from the brainstem. In simplistic terms, respiratory motor activity comprises two phases, inspiration (uptake of oxygen, O_2_) and expiration (release of carbon dioxide, CO_2_). Breathing is not rigid, but instead highly adaptable to external and internal physiological demands of the organism. The neurons that generate, monitor, and adjust breathing patterns locate to two major brainstem structures, the pons and medulla oblongata. Extensive research over the last three decades has begun to identify the developmental origins of most brainstem neurons that control different aspects of breathing. This research has also elucidated the transcriptional control that secures the specification of brainstem respiratory neurons. In this review, we aim to summarize our current knowledge on the transcriptional regulation that operates during the specification of respiratory neurons, and we will highlight the cell lineages that contribute to the central respiratory circuit. Lastly, we will discuss on genetic disturbances altering transcription factor regulation and their impact in hypoventilation disorders in humans.

## Introduction

In vertebrates, the developing brainstem generates an enormous diversity of neuron types that control bodily homeostasis and process multiple modalities of sensory information (Nieuwenhuys, 2011; Puelles et al., 2013; Venkatraman et al., 2017). These neurons vary not only in their morphological, chemical, and electrophysiological properties, but also in their connectivity patterns that allow them to form elaborate circuits, such as those required to generate, monitor, and adjust breathing to meet various external and internal physiological demands. How this neuronal diversity emerges during development and how it contributes to functional circuits has been intensively investigated by many generations of neuroscientists, as well as by cellular, molecular, and developmental biologists.

The work of these brainstem enthusiasts has already revealed that neuronal diversity in this brain region depends on the temporal and spatial patterning of local neural progenitors (Gray, 2008, 2013; Alexander et al., 2009; Hernandez-Miranda et al., 2017a; van der Heijden and Zoghbi, 2020; Isik and Hernandez-Miranda, 2022). This patterning is first achieved by early diffusible cues that impose an anterior-posterior identity, and subsequently by other morphogens that provide a distinctive dorsal-ventral molecular signature to progenitor cells. This means that distinct progenitor cells can be distinguished primarily based on their differential expression of numerous transcription factors that commit them to generate specific neuron types. Most transcription factors normally expressed in progenitor cells are later silenced in their progeny, which allows for the postmitotic maturation of the differentiated neurons. At the same time, each neuron type can also be characterized by the expression of particular sets of transcription factors that form part of their cell physiology and identity.

In this review, we aim to summarize our current knowledge on the transcriptional programs that allow for the speciation of brainstem respiratory neurons. We will first present a general overview of the anterior-posterior and dorsal-ventral patterning of the developing brainstem as an entry point to understand its neuronal diversity. Next, we will focus on the specification of three large groups of neurons that play key roles in respiration: (i) the pontine groups, (ii) the dorsal medullary respiratory column, and (iii) the ventral medullary respiratory column. These neurons locate to the pons and the medulla oblongata where they perform a variety of functions, such as respiratory rhythm generation, respiratory modulation, and tissue gas monitoring. Lastly, we will discuss some genetic disturbances that affect breathing and respiratory neuron specification.

## Anterior to posterior patterning of the developing brainstem

**FIGURE 1:**
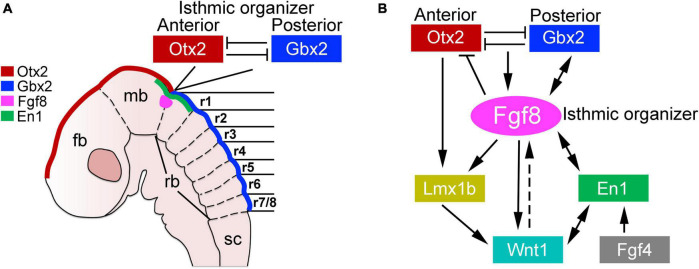
Development of the midbrain-hindbrain border. **(A)** Schema illustrating the molecular and anatomical establishment of the anterior and posterior brain regions by the expression of *Otx2* and *Gbx2*, respectively, in a developing mouse embryo (between E8-E8.5). The forebrain (fb), midbrain (mb), rhomboencephalon (rb), and spinal cord (sc) are indicated. The transient morphological segments of the rhombencephalon (rhombomeres, r) are also illustrated. **(B)** Molecular networks acting in the midbrain–hindbrain border for the establishment of the isthmic organizer (see the text). This figure is adapted from our previous publication Lowenstein et al. (2022) that was published under the terms of the Creative Commons Attribution License, which permits unrestricted use and redistribution, provided that the original author and source are credited (https://creativecommons.org/licenses/by/4.0/).

Brainstem development is an evolutionary conserved process that begins with the specification of the mesencephalon (midbrain) and rhombencephalon (hindbrain) by the isthmic organizer. This organizer is located at the midbrain-hindbrain border and produces diffusible morphogens (particularly Wnt1 and Fgf8 ligands) that act directly on the neighboring nervous tissue to impose midbrain and hindbrain cell fates (Zervas et al., 2004; Dworkin et al., 2012; Gibbs et al., 2017; Gutzman et al., 2018; Hidalgo-Sanchez et al., 2022). Various transcription factors are differentially expressed anterior and posterior to the isthmic organizer, such as the antagonistic homeodomain factors *Otx2* (anterior) and *Gbx2* (posterior), whose expression defines the rostral and caudal regions of the developing central nervous system (Figure 1A; Millet et al., 1996, 1999; Broccoli et al., 1999; Hidalgo-Sanchez et al., 1999; Joyner et al., 2000; Katahira et al., 2000; Di Giovannantonio et al., 2014). Alterations in the expression of *Otx2* and *Gbx2* are catastrophic for early brainstem development. For instance, the ablation of *Otx2* and its closely related family member *Otx1* results in the loss of midbrain tissue, which becomes re-specified into more rostral hindbrain-like regions, such as the cerebellum (Acampora et al., 1997; Suda et al., 1997; Meyers et al., 1998). Similarly, the misexpression of *Gbx2* disrupts the correct positioning of the isthmic organizer and the development of rostral hindbrain (Acampora et al., 1997; Wassarman et al., 1997; Millet et al., 1999; Puelles et al., 2003; Waters and Lewandoski, 2006).

The regulation of both *Otx2* and *Gbx2* largely depends on a complex molecular network that centers on the instructive signals of Fgf8 (Figure 1B; Crossley et al., 1996; Meyers et al., 1998; Martinez et al., 1999; Matsunaga et al., 2002; Chi et al., 2003; Prakash et al., 2006). About embryonic (E) 7.5 in mice, *Fgf8* expression is activated by the diffusible ligand Fgf4 (from the notochord) that directly induces the presumptive midbrain tissue to express the transcription factor *En1*, which in turns activates *Fgf8* expression in the isthmic organizer (Shamim et al., 1999). Two *Fgf8* isoforms appear to differentially act on the specification of the midbrain and rostral hindbrain. *Fgf8a* safeguards midbrain identities, while *Fgf8b* directs rostral hindbrain development. It is important to note that the expression of both isoforms is indispensable for the development of the midbrain and the hindbrain (Lee et al., 1997; Liu et al., 1999, 2003; Sato et al., 2001; Guo and Li, 2007). Notably, the duration of *Fgf8* expression in the isthmic organizer is crucial, as available evidence shows that its sustained expression is required to restrict *Otx2* rostral to the isthmic organizer, while simultaneously maintaining *Gbx2* caudal to it (Liu et al., 1999; Martinez et al., 1999; Sato and Joyner, 2009). In addition to controlling the expression patterns of *Otx2* and *Gbx2*, Fgf8 induces the expression of the homeodomain transcription factor *Lmx1b*, which primary function is to stabilize *Wnt1* expression in the isthmic organizer. In this regard, several studies show that the concomitant ablation of *Lmx1b* and its family member *Lmx1a* severely alters the specification of the hindbrain, which adopts a “spinal cord-like” fate in *Lmx1a* and *Lmx1b* double mutant mice (Mishima et al., 2009; Su et al., 2014; Glover et al., 2018; Chizhikov et al., 2021). Thus, the establishment of the midbrain–hindbrain border, a prerequisite for brainstem development, relies on a complex molecular network of various transcription factors and signaling cascades (briefly summarized in Figure 1B).

**FIGURE 2:**
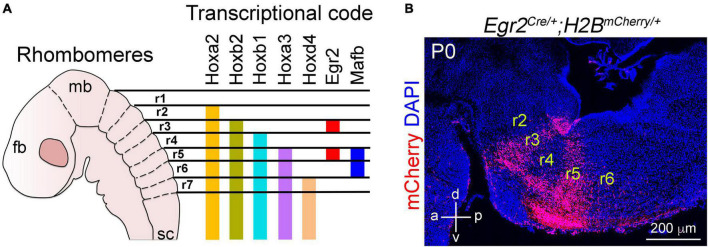
Anterior to posterior patterning of the developing brainstem. **(A)** Schema illustrating the segmentation of the brainstem into rhombomeres (r) and selected transcription factors differentially express within these rhombomeres. For more details on Hox gene expression in the developing hindbrain please see Alexander et al. (2009). **(B)** A sagittal section of the mouse brainstem stained with antibodies against mCherry and counterstained with DAPI. The section is taken from *Egr2*^*Cre/*+^;*H2B*^*mCherry/*+^ mice at birth (P0). In these mice, only r3 and r5 neural derivatives express the nuclear mCherry protein after Cre mediated recombination. This figure is adapted from our previous publication Isik and Hernandez-Miranda (2022) in Handbook of Clinical Neurology, Chapter 5, entitled Early development of the breathing network, published by Elsevier Books. The license number 5392080585902 between Hernandez-Miranda, Charite Universitätsmedizin Berlin and Elsevier allows us to reuse it in a journal/magazine.

Soon after the establishment of the isthmic organizer, the hindbrain undergoes a series of morphological changes that transiently divide it into seven or eight smaller segments called rhombomeres, from which the cerebellum, pons and medulla oblongata emerge (Bally-Cuif and Wassef, 1995; Lumsden and Krumlauf, 1996). In mice, these segments are recognizable at E8.5, whereas in humans they appear by E29 (Figure 1A; Lumsden and Krumlauf, 1996). Each of these rhombomeres develops a specific set of cellular and molecular features that distinguishes them from the adjacent nervous tissue. Although still unclear, recent anatomical studies indicate that some of rhombomeres might be further regionalized according to the expression of some patterning genes (Tomas-Roca et al., 2016; Watson et al., 2017, 2019; Hirsch et al., 2021). An interesting trait in the “rhombomerization” of the hindbrain is the differential expression of the Hox superfamily of transcription factors, whose expression creates molecular codes that coincides with the morphological borders of each rhombomere (Figure 2; Fienberg et al., 1987; Fraser et al., 1990; Krumlauf et al., 1993; Alexander et al., 2009). One should note that these transcriptional codes are not limited to the hindbrain, as some members of the Hox family are differentially expressed in the developing spinal cord, from which the characteristic cervical, thoracic, lumbar, sacral and coccygeal levels emerge (Alexander et al., 2009). In addition to Hox genes, several other transcription factors show rhombomeric specific expression patterns during early hindbrain development, such as *Pax2* in rhombomere 1, *Meis2* in rhombomeres 2 and 3, *Egr2* (formely known as *Krox20*) in rhombomeres 3 and 5, and *MafB* (previously called *Kreisler*) in rhombomeres 5 and 6 (Figure 2; Seitanidou et al., 1997; Rowitch et al., 1999; Bouchard et al., 2000, 2005; Voiculescu et al., 2001; Choe et al., 2002; Manzanares et al., 2002; Giudicelli et al., 2003; Aragon et al., 2005; Stedman et al., 2009). The timely expression of hox genes, as well as the above mentioned transcription factors, is essential for the correct development of each rhombomere and significantly depends on active derivatives of vitamin A, such as retinoic acid (Gavalas and Krumlauf, 2000). Indeed, dietary deficiencies in vitamin A produce gross disturbances in hindbrain “rhombomerization” that can lead to the complete absence of caudal rhombomeres, as manifested by the loss of specific genes and neuron types normally produced in these regions (Gavalas and Krumlauf, 2000; Glover et al., 2006; Kam et al., 2012; Addison et al., 2018). Thus, early brainstem development relies on the function of diffusible ligands (i.e., Fgf8 and retinoids) that determine its anterior-posterior identity.

## Dorsal to ventral patterning of the developing brainstem

**FIGURE 3:**
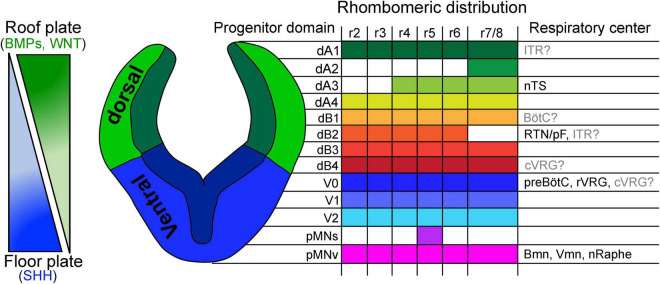
Dorsal to ventral patterning of the developing brainstem. Left, schema illustrating concentration gradients formed by the diffusion of bone morphogenic proteins (BMPs)/WNTs and Sonic hedgehog (SHH) morphogens produced by the roof and floor plate, respectively. Right, schema illustrating a transverse section of the developing hindbrain and the patterning of 13 progenitor domains along its dorsal-ventral axis. These progenitors emerge from the differential action of BMP/WNT/SHH concentration gradients. The rhombomeric distribution of each progenitor domain and contribution to brainstem respiratory centers are indicated. Of note, the origin of the intertrigeminal region (ITR), Bötzinger complex (BötC) and caudal ventral respiratory group (cVRG) has not been conclusively determined (see text). nTS, nucleus tractus solitarius; RTN/pF, retrotrapezoid/parafacial nucleus; preBötC, preBötzinger complex; rVRG, rostral ventral respiratory group; Bmm, branchial motor neurons; Vmn, visceral motor neurons; nRaphe, Raphe nuclei. This figure is adapted from our previous publication Isik and Hernandez-Miranda (2022) in Handbook of Clinical Neurology, Chapter 5, entitled Early development of the breathing network, published by Elsevier Books. The license number 5392080585902 between Hernandez-Miranda, Charite Universitätsmedizin Berlin and Elsevier allows us the reuse of it in a journal/magazine.

Once the brainstem acquires its anterior-posterior identity, a second series of diffusible cues further pattern the identity of progenitor cells along its dorsal-ventral axis. During this patterning, the ventralizing Sonic hedgehog (produced by the floor plate), as well as the dorsalizing bone morphogenetic proteins and Wnt ligands (secreted by the roof plate) create concentration gradients that differentially signal onto brainstem progenitor cells (Roelink et al., 1995; Liem et al., 1997; Lee et al., 1998, 2000; Muroyama et al., 2002; Ulloa and Marti, 2010; Hernandez-Miranda et al., 2017a). These gradients create a great diversity of molecularly distinct progenitor domains that vary depending on their spatial distance from the signaling source (Figure 3 and Table 1).

**TABLE 1:** Transcription factors expressed in progenitor domains of the developing hindbrain.

Early progenitor domains	bHLH transcription factors	Homeodomain transcription factors
dA1	Olig3, Atoh1	Pax3, Msx1
dA2	Olig3, Ngn1, Ngn2	Pax3, Pax7^low^, Msx1
dA3	Olig3, Ascl1, Ngn2	Pax3, Pax6, Pax7, Gsx2
dA4	Olig3, Ascl1, Ngn2, Ptf1a	
dB1	Ascl1, Ngn2, Ptf1a	Pax3, Pax6, Pax7, Gsx1/2
dB2		Phox2b
dB3	Ascl1	Pax3, Pax6, Pax7, Gsx1/2, Dbx2
dB4	Ngn1, Ngn2	Pax3, Pax6, Pax7, Dbx2
V0	Ngn1, Ngn2	Dbx1, Dbx2, Pax6, Pax7
V1	Ngn1, Ngn2	Dbx2, Pax6, Nkx6.2
V2	Ngn1, Ngn2	Dbx2, Pax6, Nkx6.1, Nkx6.2
MNs	Olig2	Pax6
MNv	Ascl1	Phox2b (early), Nkx2.2, Nkx2.9

Depending on the rhombomere, six to eight progenitor domains can be distinguished in the dorsal (also known as the alar plate) aspect of the developing hindbrain, while four to five progenitor domains can be identified in its ventral (or basal) plate (Figure 3). Outstandingly, there exist great resemblance between the developing hindbrain and the spinal cord, in terms of progenitor domains that locate to their dorsal-ventral axis, which share similar gene expression patterns, illustrating common developmental programs that occur between these two nervous system regions (Gray, 2008, 2013; Di Bonito and Studer, 2017; Hernandez-Miranda et al., 2017a; van der Heijden and Zoghbi, 2020; Diek et al., 2022). In the dorsal-most part of the alar plate, the combinatorial expression of the basic Helix-loop-Helix (bHLH) transcription factor *Olig3* with other bHLH genes distinguishes four progenitor domains that give rise to dorsal (d) class A neurons: dA1, dA2, dA3, and dA4 (Figure 3 and Tables 1, 2 for a list of genes expressed in these progenitors and postmitotic neurons) (Muller et al., 2005; Zechner et al., 2007; Liu et al., 2008; Storm et al., 2009). Ventral to class A progenitors, the alar plate contains four more progenitor domains that collectively generate class B neurons that express and depend on the homeodomain factor *Lbx1* for their proper specification (Gross et al., 2002; Muller et al., 2002; Cheng et al., 2005; Sieber et al., 2007; Pagliardini et al., 2008). The combinatorial expression of *Lbx1* with additional homeodomain transcription factors demarcates class B neurons into four types: dB1, dB2, dB3, and dB4 (Figure 3 and Tables 1, 2). Like in the alar plate, progenitor cells of the basal plate exhibit specific molecular codes of transcription factor expression that impose distinctive identities to at least five major neuron types: ventral (V) 0, V1, and V2 interneurons as well as somatic motor and vicero/branchio motor neurons (Figure 3 and Tables 1, 2; Gray, 2008, 2013; Alaynick et al., 2011; Di Bonito and Studer, 2017; van der Heijden and Zoghbi, 2020).

**TABLE 2:** Transcription factors expressed in neuronal cell types emerging from the developing hindbrain.

Early born neuron types	Transcription factors
dA1	Pou4f1, Barh1, Lhx2, Lhx9, Evx1
dA2	Pou4f1, Lhx1, Lhx5, Foxp2
dA3	Tlx3, Phox2b, Lmx1b
dA4	Foxd3, Foxp2
dB1	Lbx1, Pax2, Lhx1, Lhx5
dB2	Lbx1, Phox2b, Atoh1
dB3	Lbx1, Tlx3, Lmx1b, Prrxl1,
dB4	Lbx1, Pax2, Lhx1, Lhx5, Wt1, bHLHb5, Dmrt3
V0	Evx1, Pax2, Lhx1/5
V1	En1, Pax2, Lhx1/5
V2	Chx10, Sox14, Sox21
PMNs	Isl1/2
PMNv	Phox2b, Isl1/2 (visceral motor neurons) Gata2, Gata3, Lmx1b and Pet1 (Raphe neurons)

**FIGURE 4:**
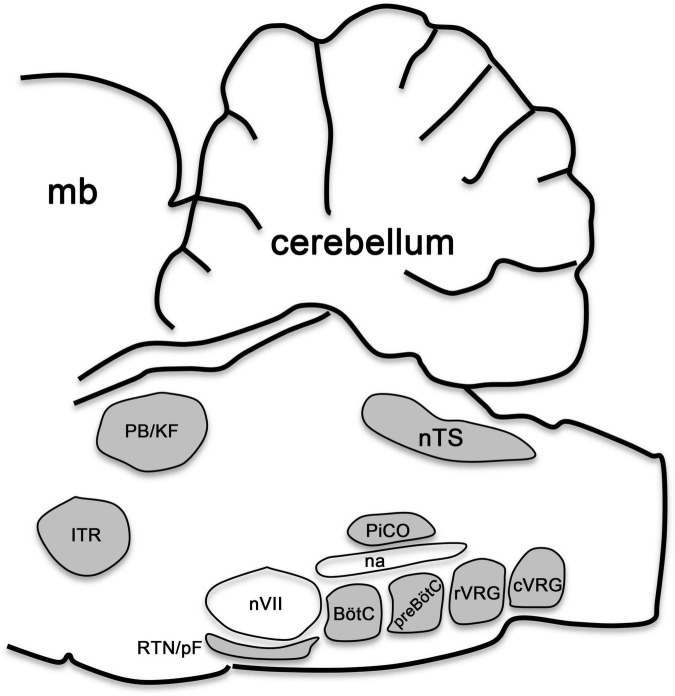
Brainstem respiratory groups. Scheme representing the sagittal view of a mouse brainstem. This scheme illustrates the location of respiratory neurons belonging to the intertrigeminal region (ITR), parabrachial/Kölliker-Fuse complex (PB/KF), nucleus tractus solitarius (nTS), retrotrapezoid/parafacial nucleus (RTN/pF), Bötzinger complex (BötC), preBötzinger complex (preBötC), postInspiratory COmplex (PiCO), rostral and caudal ventral respiratory groups (rVRG and cVRG). The cerebellum, midbrain (mb), as well as the facial (nVII) motor nucleus and the nucleus ambiguus (na) are illustrated for anatomical orientation. This figure is adapted from our previous publication Isik and Hernandez-Miranda (2022) in Handbook of Clinical Neurology, Chapter 5, entitled Early development of the breathing network, published by Elsevier Books. The license number 5392080585902 between Hernandez-Miranda, Charite Universitätsmedizin Berlin and Elsevier allows us the reuse of it in a journal/magazine.

Thus, complex networks of signaling cues pattern the developing brainstem along its anterior-posterior and dorsal-ventral axes to form a molecular grid of longitudinally and transversely distinct progenitor domains, each displaying a particular molecular code of transcription factor expression that singles out the specification of particular neuron types. In the following sections we will discuss the current knowledge of the transcriptional codes that safeguard the specification of pontine and medullary neurons that form the central respiratory circuits in the hindbrain, which collectively generate, monitor and modulate breathing (Figure 4).

## Development of the anterior respiratory groups

These respiratory groups locate to the pons and include two major structures: (i) the dorso-lateral parabrachial complex and its associated Kölliker-Fuse nucleus (for simplicity here shortened to parabrachial/Kölliker-Fuse complex) that surrounds the cerebellar peduncle, and (ii) the intertrigeminal (also known as the peritrigeminal) region that surrounds the trigeminal motor nucleus. The parabrachial/Kölliker-Fuse complex is key in the transition phase between inspiration and expiration. It is classically considered the major component of the pontine pneumotaxic center that controls the amount of air inspired in each breath by providing an off-switch for inspiration (Chamberlin and Saper, 1994; Alheid et al., 2004; Song et al., 2006). The function of the intertrigeminal region is yet to be defined, although available evidence suggests that this region might be an anti-apneic breathing center (Radulovacki et al., 2003, 2004a,b; Stoiljkovic et al., 2009; van der Heijden and Zoghbi, 2018).

Using conditional mutagenesis and lineage-tracing experiments, the group of Huda Y. Zoghbi has studied the development of these pontine groups to great effect. These studies show that both the parabrachial/Kölliker-Fuse complex and intertrigeminal region depend on the bHLH transcription factor *Atoh1* (formerly called *Math1*) for their development, as ablation of *Atoh1* results in the absence of these pontine groups in mice (Wang et al., 2005; Rose et al., 2009a,b). Recently, Van der Heijden and Zoghbi traced the rhombomeric origin of the parabrachial/Kölliker-Fuse complex to rhombomere 1, whereas they identified the intertrigeminal region to derive from rhombomere 2 (van der Heijden and Zoghbi, 2018). One should recall that rhombomere 1 development depends on the correct expression of *En1*, whereas *Hoxa2* is the most rostral Hox gene expressed in the developing hindbrain and delimits the border between rhombomeres 1 and 2 (Figures 1, 2). The importance of neurons derived from *En1*-expressing cells to respiration has not been specifically investigated, but an early report showed that *En1* null mutant mice die at birth and present with severe malformations of the cerebellum, midbrain and the parabrachial/Kölliker-Fuse complex (Wurst and Bally-Cuif, 2001). Chatonnet et al. (2007) first explored the function of *Hoxa2*-expressing neurons in respiration in the 2000s, whose work unveiled marked respiratory phenotypes and neonatal death in *Hoxa2* null mutant mice. However, the loss of numerous brainstem structures in these mutant mice precluded the identification of the particular respiratory neurons lost by the constitutive ablations of *En1* or *Hoxa2*.

In the more recent van der Heijden and Zoghbi study, the ablation of *Atoh1* from En1-expressing cells, using *En1*^*Cre/*+^;*Atoh1*^*LacZ/Flox*^ mice, leads to the absence of a recognizable parabrachial/Kölliker-Fuse complex (van der Heijden and Zoghbi, 2018). Physiologically, the absence of this respiratory group does not affect basal respiratory parameters, although spontaneous apneas and frequent sighing behavior is observed in *En1*^*Cre/*+^;*Atoh1*^*LacZ/Flox*^ mice. Notably, the specific elimination of the parabrachial/Kölliker-Fuse complex impairs respiratory chemoreflexes to hypoxia (low oxygen) and hypercarbia (high carbon dioxide). Despite the fact the parabrachial/Kölliker-Fuse complex does not sense changes in blood gases by itself, it is known to form reciprocal connections with the nucleus tractus solitarius, a center known to mediate respiratory chemoreflexes (Alheid et al., 2004, 2011; Hernandez-Miranda et al., 2017b). Thus, the loss of communication between the nucleus tractus solitarius and the parabrachial/Kölliker-Fuse complex might account for the impaired chemoreflexes observed in *En1*^*Cre/*+^;*Atoh1*^*LacZ/Flox*^ mutants. In addition, van der Heijden and Zoghbi restricted the ablation of *Atoh1* to rhombomere 2 by using a transgenic mouse line that specifically expresses Cre in rhombomere 2 derived cells (*Hoxa2*^:*CreTG*^;*Atoh1*^*LacZ/Flox*^ mice). In doing so, these scientists anatomically demonstrated the aberrant migration and defective location of intertrigeminal neurons in their conditional mutants. Physiologically, these animals show sigh-induced spontaneous apneas and smaller respiratory tidal volumes that led to a mild hypoventilation phenotype, but otherwise they are fully capable to respond to hypoxic and hypercarbic chemoreflexes. Thus, the anterior-posterior origin of the parabrachial/Kölliker-Fuse complex and intertrigeminal region has been assigned to rhombomeres 1 and 2, respectively.

During development, *Atoh1* is transiently expressed (E10.5-E13.5) in progenitor cells of the dA1 domain that encompasses rhombomere 1 (also known as the rostral or upper rhombic lip) and rhombomeres 2–7/8 (known as the caudal or lower rhombic lip) (Figure 3 and Table 1; Ben-Arie et al., 1997; Helms and Johnson, 1998; Bermingham et al., 2001; Storm et al., 2009; Lowenstein et al., 2021). Therefore, the study of van der Heijden and Zoghbi (2018) indicates that the upper rhombic lip is the bona fide origin of the parabrachial/Kölliker-Fuse complex. The progenitor domain that generates the intertrigeminal region remains to be conclusively identified. Given that intertrigeminal neurons express the homeodomain factors *Lbx1* and *Phox2b*, in addition to *Atoh1*, the source of intertrigeminal neurons could be: the dA1 (Atoh1 +) or the dB2 (Phox2b +) progenitor domain in rhombomere 2 (Wang et al., 2005; Dubreuil et al., 2009; Rose et al., 2009a,b; Hernandez-Miranda and Birchmeier, 2015; Ruffault et al., 2015; Hernandez-Miranda et al., 2017a,^2018^). Hence, either dA1-derived neurons activate *Phox2b* and *Lbx1* expression or dB2-derived (Phox2b+/Lbx1+) neurons switch on the expression of *Atoh1*. Current evidence suggests that the latter option is the correct, as retrotrapezoid neurons, a sub-population of dB2 neurons produced in rhombomere 3 or 5, originates from Phox2b + (dB2) progenitors whose progeny subsequently express *Lbx1* and *Atoh1* (Dubreuil et al., 2009; Huang et al., 2012; Hernandez-Miranda et al., 2018).

## Development of the dorsal medullary respiratory column

**FIGURE 5:**
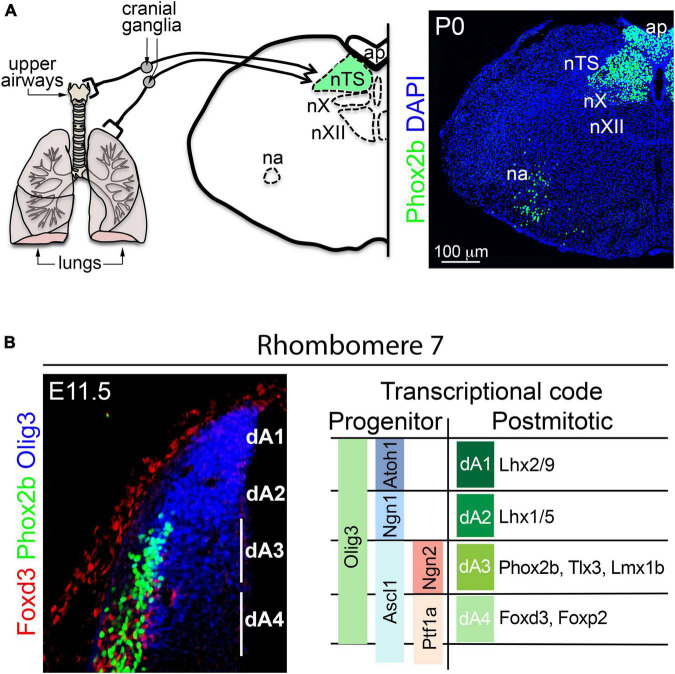
Development of the nucleus tractus solitarius. **(A)** Left, schema illustrating the relay of sensory information from the upper and lower airways to the nucleus tractus solitarius (nTS) by cranial (vagal) ganglion neurons. The nucleus ambiguus (na), area postrema (ap), nucleus vagus (nX), and the nucleus hypoglossus (nXII) are displayed as landmarks. Right, transverse section of the brainstem stained with antibodies against Phox2b and counterstained with DAPI. Phox2b expression can be observed in nTS, ap, nX and na neurons. **(B)** Left, transverse section of the dorsal rhombomere 7 stained with antibodies against Olig3, Phox2b and Foxd3 at E11.5 in mice. The expression of Olig3 encompasses the progenitor domains dA1-dA4. Phox2b and Foxd3 are differentially express in neurons emerging from dA3 and dA4, respectively. Right, schema illustrating the differential expression of transcription factors in dA progenitor cells and neurons of the dorsal rhombomere 7. This figure is adapted from our previous publication Isik and Hernandez-Miranda (2022) in Handbook of Clinical Neurology, Chapter 5, entitled Early development of the breathing network, published by Elsevier Books. The license number 5392080585902 between Hernandez-Miranda, Charite Universitätsmedizin Berlin and Elsevier allows us to reuse it in a journal/magazine. The primary data used in this figure was published in Hernandez-Miranda et al. (2017b) under the terms of the Creative Commons Attribution License, which permits unrestricted use and redistribution, provided that the original author and source are credited (https://creativecommons.org/licenses/by/4.0/).

This respiratory column contains neurons known to be critical for the regulation of inspiratory activity. Most neurons forming this respiratory column reside within the nucleus tractus solitarius (nTS, bilaterally located in the dorsal medulla oblongata), although a few neurons belonging to this column can be found in the reticular formation adjacent to the nTS. The nTS extends from the caudal level of the facial motor nucleus (at the border between rhombomeres 6/7) to the cervical spinal cord. It represents the primary entry site of peripheral viscerosensory information into the central nervous system (Figure 5A; Machado et al., 1997; Travagli, 2007; Grill and Hayes, 2009). This nucleus contains second order sensory neurons that further process and relay this information to other brain regions in the brainstem (i.e., parabrachial/Kölliker-Fuse complex), forebrain and spinal cord (Aicher et al., 1995, 1996; Alheid et al., 2011). The intermediate (at the level of the area postrema) and caudal regions of the nTS receive cardiovascular and respiratory viscerosensory afferents, while the rostral to intermediate nTS primarily receives digestive information from the vagal and glossopharyngeal nerves (Kumada et al., 1990; Vangiersbergen et al., 1992; Zoccal et al., 2014; Kawai, 2018). With respect to respiration, the intermediate nTS receives afferent information from slowly adapting pulmonary stretch receptors, whereas the caudal nTS receives afferent information from the peripheral chemoreceptors (that is the carotid bodies) and from rapidly adapting pulmonary stretch receptors (Mifflin et al., 1988; Mifflin, 1992, 1993; Machado et al., 1997; Kubin et al., 2006; Chang et al., 2015; Zhao et al., 2022). Furthermore, the nTS harbors several groups of premotor neurons that can control, for instance, the laryngeal and expiratory motor activity used in breathing-associated behaviors, such as in vocalization (Hernandez-Miranda et al., 2017b).

Histological studies traced the origin of excitatory nTS neurons to the dA3 progenitor domain (located between rhombomeres 4 and 7/8; Figure 3). This progenitor domain shares molecular traits with a progenitor domain in the spinal cord called dl3 (Muller et al., 2005; Storm et al., 2009; Hernandez-Miranda et al., 2017a). Indeed, neuronal derivatives from these progenitor domains are excitatory and seem to only vary in the expression of the transcription factors *Phox2b* (in dA3 neurons) and *Isl1* (in dI3 neurons), but otherwise they co-express the transcription factors *Pou4f1*, *Tlx3*, *Prrxl1*, and *Lmx1b* (Figures 3, 5B; Chen et al., 2001; Qian et al., 2001, 2002; Cheng et al., 2004; Muller et al., 2005; Liu et al., 2008; D’Autreaux et al., 2011). For over a quarter century the group of Christo Goridis and Jean-François Brunet has characterized the critical roles of *Phox2a* and *Phox2b* in visceral nervous system development. These homeobox transcription factors are necessary for the development of central and peripheral noradrenergic neurons, parasympathetic and sympathetic ganglia, branchial, and visceral motor neurons, as well as primary and secondary viscerosensory neurons (Morin et al., 1997; Pattyn et al., 1997, 1999, 2000a,2000b,^2006^; Fode et al., 1998; Dauger et al., 2003; D’Autreaux et al., 2011; Espinosa-Medina et al., 2016). *Phox2b* is critical for the specification of excitatory nTS neurons, and its mutation precludes the formation of this brainstem center in mice (Pattyn et al., 1999; Dauger et al., 2003; Hernandez-Miranda et al., 2017b). In addition, most excitatory nTS neurons co-express the transcription factor *Tlx3* (previously known as *Rnx*) that seems to stabilize and maintain the expression of *Phox2b* in nTS neurons (Qian et al., 2001; Dauger et al., 2003; Storm et al., 2009; Hernandez-Miranda et al., 2017b).

In the developing hindbrain, the precise identity of dA3 progenitor cells is determined by the co-expression of the bHLH transcription factors *Olig3*, *Ascl1* and *Ngn2* (Figure 5B; Pattyn et al., 2006; Liu et al., 2008; Storm et al., 2009; Hernandez-Miranda et al., 2017a). In addition to excitatory nTS neurons, dA3 progenitors also generate other excitatory neurons that include: area postrema neurons (associated with vomiting reflexes) and caudal (nor)adrenergic neurons (the baroreflex-associated A1 and A2 groups) (Qian et al., 2001; Dauger et al., 2003; Pattyn et al., 2006; Storm et al., 2009; Zhang et al., 2021). Progenitors in the dA3 domain generate these neuron types in a temporal order in which (nor)adrenergic neurons are generated first, followed by excitatory nTS neurons and lastly area postrema neurons (Hernandez-Miranda et al., 2017b). Although most nTS neurons are excitatory, a substantial amount of inhibitory neurons also reside within this nucleus. Inhibitory nTS neurons depend on *Lbx1* and appear to derive from the dB1 or dB2 progenitor domains (Hernandez-Miranda et al., 2018). The rhombomeric origin of the nTS has not been directly investigated, incidental evidence, however, suggests that it primarily originates from rhombomeres 7 & 8, as the ablation of the transcription factor *MafB*, which severely affects the development of rhombomeres 5 and 6, does not significantly interfere with nTS development (Blanchi et al., 2003).

## Development of the ventral medullary respiratory column

Several anatomical and physiological distinct groups of respiratory-related neurons have been identified in the ventral medulla oblongata, which form the ventral medullary respiratory column, these groups include: (a) the retrotrapezoid/parafacial nucleus, (b) the Bötzinger complex, (c) the preBötzinger complex, (d) the PiCo complex (dorsal to the Bötzinger and preBötzinger complexes), (e) the rostral ventral and caudal respiratory groups, as well as (f) the raphe obscurus (Figure 4). Neurons within these groups can either project onto respiratory premotor neurons or themselves act as premotor neurons to regulate the motoric behavior associated with breathing. Interestingly, three of these groups exhibit intrinsic rhythmic activity during prenatal development: (i) the parafacial nucleus, (ii) the preBötzinger complex and (iii) a newly identified PostInspiratory COmplex (PiCo), and are believed to be central for the generation of the respiratory rhythm (Smith et al., 1991; Onimaru and Homma, 2003; Anderson et al., 2016; Del Negro et al., 2018; Ramirez and Baertsch, 2018). Except for the recently identified PiCo complex, the development of all other ventral respiratory groups has been studied.

### Development of the retrotrapezoid/parafacial nucleus

**FIGURE 6:**
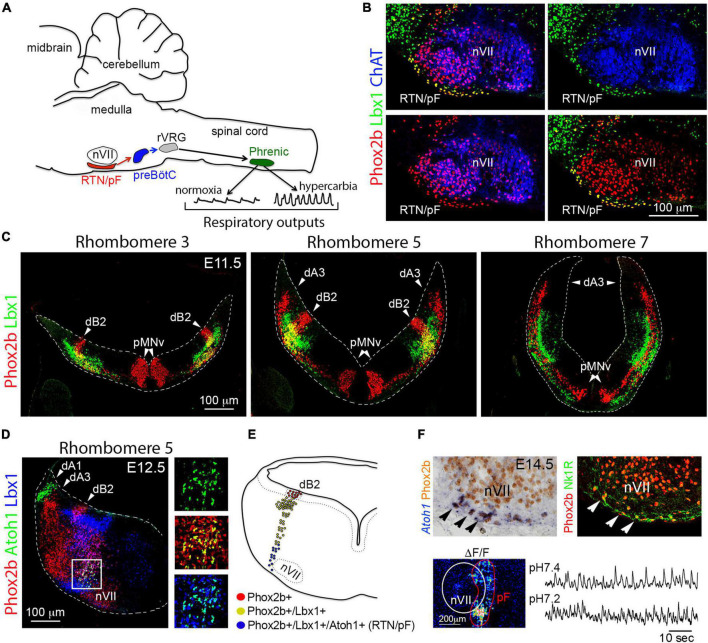
Development of the retrotrapezoid/parafacial nucleus. **(A)** Schema representing the location and function of retrotrapezoid/parafacial nucleus (RTN/pF) in mice. Under normal levels of oxygen (normoxia), the activity of the preBötzinger complex (preBötC) controls the firing rate of neurons in the phrenic motor nucleus (Phrenic) by sending signals *via* premotor neurons located in the rostral ventral respiratory group (rVRG). Phrenic motor neurons control diaphragm activity. Under hypercarbia (high levels of PCO_2_) RTN/pF neurons increase their firing rate: this adjusts the activity of preBötzinger complex and eventually the firing rate of the phrenic motor neurons, which in turn increases respiration **(B)** Histological characterization of RTN/pF in newborn mice (P0). RTN/pF neurons co-express Phox2b and Lbx1 but not choline-acetyltransferase (ChAT), which distinguishes them from facial motor neurons (nVII) that express Phox2b and ChAT but not Lbx1. **(C)** Histological characterization of dB2 neurons across the indicated rhombomeres (r). dB2 neurons co-express Phox2b and Lbx1 at E11.5 in mice. This molecular signature distinguishes them from the dorsal (dA3 neurons) and ventral (pMNv) cells that express Phox2b but not Lbx1. Note that dB2 neurons emerge from r2 to r6, whereas dA3 neurons originate in r4 to r7/8 and pMNv cells can be found from r2-r7/8 (see text). **(D)** Transverse section of a E12.5 mouse embryo stained with antibodies against Phox2b, Atoh1, and Lbx1. Note that a subset of dB2 neurons (magenta cells) activate the expression of Atoh1 as they reach the facial (nVII) motor nucleus (boxed area). The boxed area is illustrated at the right with different combinations of the fluorescent signals. **(E)** Schema depicting the development of RTN/pF neurons. Phox2b + dB2 progenitor cells differentiate an initiate the expression of Lbx1 in neurons. A subset of Phox2b + /Lbx1 + (dB2) neurons ventrally migrates and activates the expression of Atoh1 as they reach the facial (nVII) motor nucleus. It is the co-expression of Phox2b + /Lbx1 + /Atoh1 + what defines the molecular signature of RTN/pF neurons. **(F)** Upper panels, by E14.5 in mice, RTN/pF neurons (arrowheads) settle underneath the facial motor (nVII) nucleus and express additional marker, such as neurokinin 1 receptor (Nk1R). Lower panels, ventral hindbrain view of a E14.5 wholemount brainstem preparation centered on the facial (nVII) motor nucleus showing Ca2 + green-1AM fluorescence changes (ΔF/F) of parifacial nucleus (pF) activity in physiological (7.4) and low (7.2) pH. This figure is adapted from our previous publication Isik and Hernandez-Miranda (2022) in Handbook of Clinical Neurology, Chapter 5, entitled Early development of the breathing network, published by Elsevier Books. The license number 5392080585902 between Hernandez-Miranda, Charite Universitätsmedizin Berlin and Elsevier allows us to reuse it in a journal/magazine. The primary data used in this figure was published in Hernandez-Miranda and Birchmeier (2015) and Hernandez-Miranda et al. (2018) under the terms of the Creative Commons Attribution License, which permits unrestricted use and redistribution, provided that the original author and source are credited (https://creativecommons.org/licenses/by/4.0/).

The retrotrapezoid and parafacial nuclei are two small groups of excitatory neurons that are located ventro-laterally to the facial motor nucleus. Whether these groups are genuinely distinct remains to be conclusively determined. However, recent evidence seems to suggest that they are indeed physiologically distinct neuronal populations (Huckstepp et al., 2015, 2016, 2018; Korsak et al., 2018; Zoccal et al., 2018). From an anatomical point of view, retrotrapezoid neurons locate ventrally to the facial motor nucleus and are long known to contain central respiratory chemoreceptor neurons; that is, acid-activated neurons that maintain constant levels of arterial PCO_2_ (Figures 6A,B,F). For more details on the physiology of retrotrapezoid neurons and other central respiratory chemoreceptor neurons, we refer to the excellent work of Nattie and Li (2012), Guyenet and Bayliss (2015, 2022), Guyenet et al. (2019) and Lopez-Barneo (2022). On the other hand, neurons of the parafacial nucleus are located lateral to the facial motor nucleus. They are thought to control active expiration, a particular type of expiration that is produced when high metabolic demands induce an increase in respiration (Fortin and Thoby-Brisson, 2009; Thoby-Brisson et al., 2009; Bayliss et al., 2015; Huckstepp et al., 2015, 2018; Korsak et al., 2018; Pisanski and Pagliardini, 2019).

In 2009, Dubreuil et al. (2009) identified that virtually all retrotrapezoid and parafacial neurons originate from *Egr2*-expressing cells (that is from rhombomeres 3 and/or 5; see Figure 2). Furthermore, they identified their distinctive molecular signature, namely their co-expression of *Phox2b*, *Lbx1*, and *Atoh1* (Figure 6B). Ablation of each of these genes leads to the anatomical absence (*Phox2b* and *Lbx1* mutant mice) or aberrant location (*Atoh1* mutants) of retrotrapezoid and parafacial neurons, as well as to the loss of the hypercarbic reflex (the natural acceleration of breathing in response to increasing PCO_2_ levels) and neonatal death (Wang et al., 2005; Pagliardini et al., 2008; Dubreuil et al., 2009; Rose et al., 2009a,b; Hernandez-Miranda et al., 2018). The dB2 progenitor domain generates retrotrapezoid and parafacial neurons and is exclusively located between rhombomeres 2 and 6 (Figures 3, 6C). dB2 progenitor cells express *Phox2b* and their postmitotic progeny co-express *Lbx1* in addition to *Phox2b* (Figure 6C). A small number of dB2 (Lbx1 + /Phox2b +) neurons migrate ventrally toward the facial motor nucleus and activate the expression of *Atoh1* during their migration (Figures 6D,E). The expression of *Atoh1* seems to be essential for the migration and maturation of retrotrapezoid and parafacial nucleus neurons (Huang et al., 2012; Ruffault et al., 2015; Hernandez-Miranda et al., 2018). As retrotrapezoid and parafacial neurons mature throughout postnatal life, and for reasons yet to be identified, these neurons silence *Lbx1* and *Atoh1*, but retain *Phox2b*. Several studies show that interfering with the specification of retrotrapezoid and parafacial neurons does not compromise neonatal or postnatal survival in mice. However, the loss of these neurons leads to a range of hypoventilation behaviors and the loss of the hypercarbic reflex in neonatal mice (Ramanantsoa et al., 2011; Huang et al., 2012; Ruffault et al., 2015; Hernandez-Miranda et al., 2018). Notably, transgenic mice lacking retrotrapezoid and parafacial neurons are able to recover some of the ventilatory responses to hypercarbia in adult life (Ramanantsoa et al., 2011; Huang et al., 2012; Ruffault et al., 2015; Hernandez-Miranda et al., 2018). How these animals gain the ability to respond to hypercarbia in adulthood is currently unknown, but this indicates that other chemoreceptor cells, either in the central or peripheral nervous system, can compensate for the loss of retrotrapezoid and parafacial neurons in the adult life.

### Development of the Bötzinger complex

The major inhibitory component of the ventral respiratory column is the Bötzinger complex that uses GABA and glycine as its primary neurotransmitters (Shao and Feldman, 1997; Schreihofer et al., 1999; Song et al., 2001). Classic studies showed that the electrophysiological and pharmacological activation of Bötzinger neurons strongly inhibits inspiration and that Bötzinger neurons display decrementing postinspiratory or augmenting firing patterns during expiration (Bongianni et al., 1988; Gang and Lei, 1996). These neurons mutually interact with the preBötzinger complex to regulate the respiratory rhythm (Bryant et al., 1993; Tian et al., 1998; Ezure et al., 2003a,b,c). The Bötzinger complex innervates all other brainstem respiratory neurons and projects to spinal premotor and motor phrenic neurons (Otake et al., 1988; Jiang and Lipski, 1990; Douse and Duffin, 1992; Tian et al., 1998; Smith et al., 2007).

The precise rhombomeric origin and progenitor domain from which the Bötzinger complex develops has not yet been fully investigated. However, circumstantial evidence might indicate that this respiratory group develops from the *Lbx1*-lineage. Indeed, a lineage tracing study by Pagliardini et al. (2008) revealed that virtually all GABAergic and glycinergic neurons found in the anatomical region where the Bötzinger complex resides have a history of *Lbx1* expression, ablation of which results in the absence of GABAergic and glycinergic neurons in this area (Pagliardini et al., 2008). Mature “Bötzinger” neurons lose the expression of *Lbx1* but can be recognized by the expression of *Pax2*, *GABA* and other glycinergic markers (Pagliardini et al., 2008). As abovementioned, *Lbx1* is key for the specification of four distinct neuron types, two of which express *Pax2* and are GABAergic and glycinergic in nature: dB1 and dB4 neurons (Figure 3 and Table 2). In *Lbx1* null mutant mice, *Pax2* expression seems to be uniquely lost from the dB1 domain (that extends from rhombomeres 2–7/8), which might suggest this region as the possible source of Bötzinger neurons (Pagliardini et al., 2008). However, more research is necessary to clearly define the developmental origin of the Bötzinger complex.

### Development of the preBötzinger complex

**FIGURE 7:**
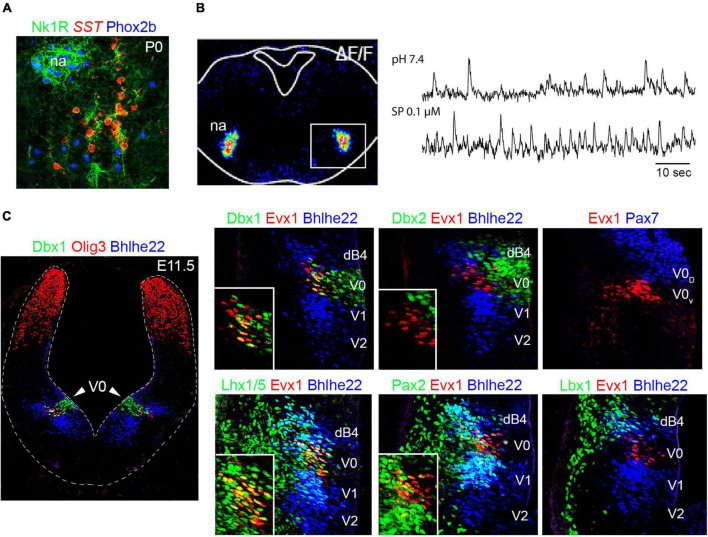
Development of the preBötzinger complex. **(A)** The neurons of the preBötzinger complex locate ventral to the nucleus ambiguus (na) and can be distinguished by the co-expression of somatotatin (SST) and neurokinin 1 receptor (Nk1R) in mice at birth (P0). Please note that preBötzinger neurons co-express SST and Nk1R but not Phox2b, while motor neurons of the nucleus ambiguus co-express Nk1R and Phox2b but not SST. **(B)** Left, transverse section of a mouse hindbrain at E14.5 showing Ca2 + green-1AM fluorescence changes (ΔF/F) of preBötzinger complex (boxed area) intrinsic activity. Right, traces illustrating bursts of preBötzinger complex activity in physiological pH or after substance p (SP) treatment, which accelerates their firing. **(C)** Left, transverse section of a E11.5 mouse brainstem at rhombomere 7. The section was stained with antibodies against Dbx1 (green) Olig3 (red) and Bhlhe22 (blue). Note that the V0 progenitor domain can be distinguished by the expression of Dbx1. Right, histological characterization of the V0 progenitor domain and the neurons that emerge from it, using antibodies against Dbx1, Dbx2, Evx1, Pax7, Lhx1/5, Pax2 and Lbx1. Note the Dbx1 + (V0) progenitor domain generates Evx1 + neurons that co-express in addition Lhx1/5 and Pax2 but not Lbx1 (see also Tables 1, 2 and text for more details). The expression of Bhlhe22 allows the distinction of the dB4 and V1/V2 domains that sandwich the V0 progenitor domain. A fraction of Dbx1-derived neurons will differentiate into the preBötzinger complex (see text). Insets in the main photographs illustrate the co-expression of Evx1 + cells with the indicated markers. This figure is adapted from our previous publication Isik and Hernandez-Miranda (2022) in Handbook of Clinical Neurology, Chapter 5, entitled Early development of the breathing network, published by Elsevier Books. The license number 5392080585902 between Hernandez-Miranda, Charite Universitätsmedizin Berlin and Elsevier allows us to reuse it in a journal/magazine. The primary data used in this figure was published in Hernandez-Miranda et al. (2017b) under the terms of the Creative Commons Attribution License, which permits unrestricted use and redistribution, provided that the original author and source are credited (https://creativecommons.org/licenses/by/4.0/).

In the early 1990’s, the work of Jeffrey C. Smith and Jack L. Feldman identified the preBötzinger complex as essential for generating the respiratory rhythm in mammals (Figures 7A,B; Smith et al., 1991). Later studies uncover some molecular markers such as the neurokinin 1 receptor and somatostatin to be expressed by preBötzinger neurons (Smith et al., 1991, 2007; Gray et al., 1999, 2001). How the preBötzinger complex generates the respiratory rhythm is currently the subject of intense investigation and outside the scope of this review, but we recommend the reader the excellent reviews by the groups of Anderson and Ramirez (2017), Ramirez and Baertsch (2018), Rubin and Smith (2019), Ramirez et al. (2022) and Smith (2022).

In 2010, two independent groups defined the V0 progenitor domain (in the basal plate) as the source of the preBötzinger complex (Bouvier et al., 2010; Gray et al., 2010). V0 progenitor cells share molecular traits with the p0 progenitor domain in the ventral spinal cord and are characterized by the expression of Dbx1 and Dbx2, the former is, however, unique for V0 and p0 progenitor cells (Figure 7C and Table 1; Pierani et al., 1999, 2001). Both Bouvier et al. (2010) and Gray et al. (2010) used *Dbx1*^LacZ^** mice, which express the reporter protein beta-galactosidase under the control of the *Dbx1* promoter, to lineage trace the preBötzinger complex and demonstrated that all the excitatory and rhythmically active neurons in this region derived from the V0 progenitor domain. Neurons emanating from this domain express the transcription factor *Evx1*, which distinguishes them from the surrounding dB4 and V1 neurons that express *Lbx1* and *En1*, respectively (Figure 7C and Table 2; Pierani et al., 1999, 2001). Interestingly, V0 neuronal derivatives destined to populate the preBötzinger complex are correctly generated and reach the preBötzinger complex area in *Dbx1* null mutant (*Dbx1*^LacZ/LacZ^**) mice, but these cells do not exhibit intrinsic rhythmicity and fail to adopt their mature molecular identity, as determined by the lack of neurokinin 1 receptor or somatostatin expression in these cells (Bouvier et al., 2010).

Expression of the transcription factor *Pax7* molecularly distinguishes two V0 progenitor subdomains: V0 dorsal (V0_D_; Pax7 +) and V0 ventral (V0_V_; Pax7-) (Figure 7C). In Bouvier et al. (2010) the authors walked an extra mile and ablated *Dbx1* from the V0_D_ domain, using *Pax7*^Cre^*;Dbx1*^LacZ/Flox^** mice, to elucidate which of these two subdomains generate the preBötzinger complex. This elegant experiment found an anatomically, molecularly and physiologically intact preBötzinger complex, demonstrating that this respiratory center emerges from the V0_V_ subdomain. More recent studies have exploited this developmental knowledge to selectively activate, silence, or ablate Dbx1-derived preBötzinger neurons, resulting in respiratory changes that enhance, depress, or halt breathing in adult mice, respectively (Wang et al., 2014; Vann et al., 2016, 2018).

Even though the anterior-posterior origin of the preBötzinger complex has not yet been identified, an early study indicates that it emerges from the most posterior rhombomeres and partly from rhombomere 6 (*MafB* +). In this context, a study by Blanchi et al. (2003) showed that *MafB* null mutant mice rarely breathe at birth and display limited phrenic nerve activity (Blanchi et al., 2003). Histologically, *MafB* null mutants have a significant, but not complete, loss of preBötzinger neurons (Blanchi et al., 2003). Since *Egr2* null mutant mice display breathing rhythmicity and can survive for about a day after birth (Chatonnet et al., 2007), it is likely that V0 progenitors in rhombomere 6 generate a fraction of preBötzinger neurons that is complemented by V0 progenitors in rhombomeres 7/8.

### Development of the rostral and caudal ventral respiratory groups

The preBötzinger complex controls breathing by activating premotor neurons that in turn regulate multiple brainstem (e.g., hypoglossal or vagal) and spinal cord (phrenic) motor neurons. Caudal to the preBötzinger complex, two sets of respiratory premotor neurons are associated with inspiratory and expiatory motor activity: the rostral (rVRG) and caudal (cVRG) ventral respiratory groups, respectively (Smith et al., 2013). A recent study from the group of Gilles Fortin identified that excitatory rVRG neurons have a history of *Dbx1* expression and, as such, to originate from the V0 progenitor domain (Wu et al., 2017). Even though both preBötzinger neurons and rVRG neurons express *Slc17a6* (vGlut2) and *Pax2*, only the former expresses neurokinin 1 receptor and somatostatin (Bouvier et al., 2010; Gray et al., 2010; Wu et al., 2017). These molecular differences could be explained by their distinct rhombomeric origins and/or by their generation in distinct V0 progenitor subdomains. The development of cVRG neurons has not been yet addressed, but some Dbx1-derivaties neurons can be observed caudal to the rVRG, suggesting that cVRG neurons are also generated from the V0 progenitor domain (Gray, 2013; Wu et al., 2017). An alternative source of cVRG neurons could be the dB4 domain, whose derivatives co-express *Lbx1* and *Wt1* (Table 2). Indeed, a recent study reported the presence of GABAergic (*Wt1* +) neurons in the cVRG region (Schnerwitzki et al., 2020).

### Development of respiratory serotonergic neurons

The monoamine neurotransmitter serotonin has long been implicated in the control of respiration. Brain serotonin is produced by nine distinct groups of cells (called raphe nuclei), all of which are in the brainstem. Among the distinct raphe nuclei, several lines of research indicate that the midline located raphe obscurus in the medulla oblongata is an important component of the central respiratory chemoreceptor circuit (Jacobs et al., 2002). First, the *en masse* inhibition of serotonergic neurons (Ray et al., 2011), or the targeted inhibition of raphe obscurus neurons (Brust et al., 2014), significantly impairs the chemoreflex to hypercarbia in mice. Second, the optogenetic activation of these raphe neurons accelerates respiration in conscious and anesthetized rodents (Depuy et al., 2011). Lastly, mice genetically engineered to lack raphe neurons display dulled chemoreflexes to hypercarbia (Hodges et al., 2008, 2009; Buchanan and Richerson, 2010).

The ventral-most progenitor domain (termed as pMN; Figure 3) in the developing hindbrain initially generates motor neurons, and later all brainstem serotonergic neurons. Due to its proximity to the floor plate, the pMN domain is under the direct influence of Sonic Hedgehog signaling (Marti et al., 1995; Chiang et al., 1996). Molecularly, this progenitor domain is subdivided into a dorsal subdomain (pMNs; expressing the transcription factors *Pax6* and *Olig2*) and a ventral subdomain (pMNv; expressing the transcription factors *Nkx2.2*, *Nkx2.9*, and *Phox2b*) (Figure 3 and Table 1). Detailed histological and genetic analyses showed that the pMNs subdomain generates somatic motor neurons (i.e., hypoglossal motor neurons), whereas the pMNv subdomain generates branchial (e.g., facial motor neurons) and visceral (i.e., nucleus ambiguous) motor neurons (Briscoe et al., 1999; Pattyn et al., 2003). Pioneer studies by Briscoe and colleagues showed that pMNv progenitors first generate branchio/viscero motor neurons before E11.5 in mice, and then serotonergic neurons (Briscoe et al., 1999; Pattyn et al., 2003). One should note that except for rhombomere 4, the pMNv progenitor domain in all other rhombomeres contributes to raphe neurons. pMNv progenitor cells of rhombomere 4 are known to generate a large group of branchial (facial) motor neurons and to have an unusual prolonged expression of *Phox2b* between E9.5 to E12.5, which seems to be attributable to its incapacity to generate serotonergic neurons (Pattyn et al., 2003). Indeed, the silencing of *Phox2b* expression seems to be a molecular switch in the transition of pMNv progenitor cells from first generating branchio/visceromotor neurons to later generating serotonergic neurons in the other rhombomeres (Briscoe et al., 1999; Pattyn et al., 2003, 2004). In support of this, analysis of *Nkx2.2* null mutant mice revealed an unusual extended expression of *Phox2b* within the pMNv domain, which leads to the overproduction of branchio/visceromotor neurons and the absence of serotonergic neurons (Briscoe et al., 1999; Pattyn et al., 2003, 2004). Conversely, the ablation of *Phox2b* results in the early generation of raphe neurons at the expense of branchio/visceromotor cells (Pattyn et al., 2004).

The bHLH transcription factor Ascl1 is critical for development of peripheral (i.e., enteric nervous system) and central (raphe) serotonergic cells (Blaugrund et al., 1996; Pattyn et al., 2004). In the pMNv domain, *Ascl1* is co-expressed with *Phox2b* during the genesis of branchio/visceromotor neurons and is retained by these progenitors throughout the specification of raphe cells (Pattyn et al., 2004). Mutation of *Ascl1* does not affect *Phox2b* expression nor the development of branchio/visceromotor cells, but severely interferes with the specification of raphe neurons, which are completely absent in *Ascl1* null mutant mice (Pattyn et al., 2004). The maturation of raphe neurons is regulated by several other transcription factors, such as *Gata2*, *Gata3*, *Lmx1b* and *Pet1*, of which the null mutation of *Lmx1b* or *Pet1* results in the total loss or a severe decrease (> 70%) of raphe cells, respectively (Cheng et al., 2003; Ding et al., 2003; Hendricks et al., 2003; Dosumu-Johnson et al., 2018; Okaty et al., 2020).

## Cell lineages contributing to respiratory and non-respiratory neurons

From a developmental point of view, most brainstem respiratory neurons emerge from a few molecularly defined cell-lineages and progenitor domains: (i) an *Atoh1*-lineage that contributes to the development of the parabrachial/Kölliker-Fuse complex (dA1, in rhombomere 1) and the intertrigeminal region (either dA1 or dB2, in rhombomere 2); (ii) an *Olig3/Phox2b/Tlx3*-lineage (dA3, in rhombomere 7/8) that generates the dorsal medullary respiratory column (nTS); iii) a *Phox2b/Lbx1/Atoh1*-lineage (dB2, in rhombomere 3 and/or 5) that generates the retrotrapezoid/parafacial nuclei; (iv) an *Lbx1*-lineage (presumably dB1, unknown rhombomeric origin) that produces the Bötzinger complex; v) a *Dbx1*-lineage (V0) that gives rise to the preBötzinger complex (in rhombomeres 6-7/8) and the rVRG (and possibly the cVRG, in rhombomere 7/8) groups of premotor neurons; as well as (vi) an *Nkx2.2/Ascl1/Lmx1b*-lineage that produces raphe serotonergic neurons (pMNv, across rhombomeres).

One should not forget, however, that each of these progenitor domains produce a much greater diversity of neuron types than just respiratory neurons. A good example of this is the dA1 (Atoh1 +) domain in rhombomere 1 (upper rhombic lip). This domain generates: in addition to the parabrachial/Kölliker-Fuse complex, all excitatory deep cerebellar neurons, all cerebellar granule cell progenitors, as well as all cerebellar and cochlear unipolar brush cells (Englund et al., 2006; Fink et al., 2006; Ray and Dymecki, 2009; Hernandez-Miranda et al., 2017a; van der Heijden and Zoghbi, 2018, 2020; Elliott et al., 2021; Lowenstein et al., 2021, 2022; Fritzsch et al., 2022). How these progenitors generate such a vast array of neuron types is currently being investigated and appears to depend on the temporal expression of transcription factors that act as selector genes. In this context, the co-expression of *Atoh1* with *Olig3* is critical for deep cerebellar neuron development, whereas the co-expression of *Atoh1* with *Neurod1* is essential for granule cell progenitor specification and cerebellar and cochlear unipolar brush cell development (Ben-Arie et al., 1997; Chizhikov and Millen, 2003; Gazit et al., 2004; Pan et al., 2009; Machold et al., 2011; Lowenstein et al., 2021). The selector gene for the specification of parabrachial/Kölliker-Fuse complex is presently unknown.

It might not be surprising that across rhombomeres, each progenitor domain generates different neuron types. Nonetheless, an interesting trait of these spatially segregated progenitor domains is that their shared expression of transcription factors might instruct their progeny to synaptically connect and form functional circuits. For instance, both Atoh1 + /Olig3 + (dA1) progenitors in rhombomere 7 or the pdI1 progenitors in the spinal cord (equivalent to dA1) produce second relay neurons that project to the cerebellum and synapse onto granule cells and deep cerebellar neurons that derive from rhombomere 1 dA1 (Atoh1 + /Olig3 +) progenitors (Muller et al., 2005; Liu et al., 2008; Storm et al., 2009; Hernandez-Miranda et al., 2017a; Lowenstein et al., 2021, 2022). Another example of this molecular logic could be the inferior olive-Purkinje cell circuit. Indeed, inferior olive cells that derive from Olig3 + /Ptf1a + (dA4 in rhombomere 7) progenitors send axons that synapse onto Purkinje cells that emerge from Olig3 + /Ptf1a + progenitors in the cerebellar ventricular zone in rhombomere 1 (Liu et al., 2008; Storm et al., 2009; Hernandez-Miranda et al., 2017a; Lowenstein et al., 2021, 2022). To which extent these shared transcriptional codes allow for the interconnection of the distinct neurons that form the brainstem respiratory circuit is presently unknown. However, emerging evidence shows similar developmental strategies, i.e., preBötzinger complex neurons that connect with the rVRG are both derivatives of the V0 domain (Bouvier et al., 2010; Gray et al., 2010; Wu et al., 2017). An even more conspicuous case is the *Phox2b*-lineage that generates virtually all neurons that form the central and peripheral visceral nervous system (Morin et al., 1997; Pattyn et al., 1997, 1999, 2000a, 2000b, 2003, 2004, 2006; Fode et al., 1998; Dauger et al., 2003; D’Autreaux et al., 2011; Espinosa-Medina et al., 2016; Isik and Hernandez-Miranda, 2022). The question of how these developmental strategies emerged during evolution remains to be explored.

## Conclusion

The enormous knowledge gained during the last three decades of brainstem development research now allows us to further dissect the function of respiratory neurons with unprecedented detail. Several experimental and theoretical approaches have recently taken advantage of the developmental trajectories of respiratory neurons to explore the complex character of the respiratory rhythm generator or to elucidate different components of the central chemoreceptor circuit (Depuy et al., 2011; Ray et al., 2011; Brust et al., 2014; Wang et al., 2014; Vann et al., 2016, 2018).

Many aspects of how respiratory neuron diversity emerges during development remain to be elucidated. The identification of how the brainstem generates respiratory neurons is critical to understand this complex behavior and essential for the development of new therapeutic approaches for the management of respiratory diseases. In this context, recent studies on the genetic disturbances causing congenital respiratory syndromes are currently steering our views into both the development and function of respiratory neurons. A clear example is the study of congenital central hypoventilation syndrome (CCHS, also known as Ondine’s curse; OMIM 209880). Although rare (1 in 200,000 live births), this disorder is life threatening and characterized by slow, apneic and shallow breathing (hypoventilation) while awake and respiratory arrest during sleep (Weese-Mayer et al., 2008, 2017; Sivan et al., 2019; Ceccherini et al., 2022). Frequently, CCHS patients also present with blunted responses to hypercabia and have abnormal levels of PCO_2_. Early genetic studies identified *de novo* mutations in *PHOX2B* as the most prevalent cause of CCHS (Amiel et al., 2003). Two types of *PHOX2B* mutations that cause CCHS have been identified: (i) polyalanine repeat expansions, and (ii) non-polyalanine repeat expansions that are more prevalent in severe cases of CCHS (Amiel et al., 2003; Zhou et al., 2021). CCHS patients with *PHOX2B* mutations frequently manifest Hirschsprung’s disease, revealing that genetic disturbances on *PHOX2B* can simultaneously alter the development of both the central and peripheral nervous systems (Weese-Mayer et al., 2008, 2017; Sivan et al., 2019; Ceccherini et al., 2022). *PHOX2B* is a central factor in the development and function of the visceral nervous system, whose mutation results in midterm fetal lethality (Dauger et al., 2003). Interestingly, the insertion of a frequent poly-alanine *PHOX2B* mutation (called *PHOX2B*^+*7ala*^) into the murine genome has shown that this aberrant expansion only affects a subset of *Phox2b* functions, as only one Phox2b-dependent neuron type (the retrotrapezoid nucleus) does not develop correctly in *Phox2b*^+*7ala*^ mutant mice (Dubreuil et al., 2008). More recently, the characterization of a CCHS disease-causing frameshift mutation in *LBX1* has further revealed that this respiratory disorder originates from the misspecification of dB2 neurons in mice, and that this is caused by a lack of cooperativity between *PHOX2B* and *LBX1* (Hernandez-Miranda et al., 2018).

For many years, the size and location of the brainstem was a major impediment to comprehend its physiology. With the advent of new technologies such as single-cell transcriptomics, monosynaptic viral tracing as well as opto- and chemo- genetic tools, several of the long-lasting obstacles associated with the identification and modulation of specific breathing behaviors are now amenable for scientific exploration. There is no doubt that the years to come will foster and propel our understanding of this elementary and humble animal behavior in ways that we can only now imagine.

## Author contributions

YX, KC, AA, EL, and LH-M reviewed the literature. LH-M wrote the original draft and edited it with the input from all authors. All authors contributed to the article and approved the submitted final version.
